# Evaluation of scoring systems without endoscopic findings for predicting outcomes in patients with upper gastrointestinal bleeding

**DOI:** 10.1186/s12876-017-0716-4

**Published:** 2017-12-12

**Authors:** Il-Gyu Ko, Sung-Eun Kim, Bok Soon Chang, Min Seob Kwak, Jin Young Yoon, Jae Myung Cha, Hyun Phil Shin, Joung Il Lee, Sang Hyun Kim, Jin Hee Han, Jung Won Jeon

**Affiliations:** 1 Department of Physiology, College of Medicine, Kyung Hee University, Seoul, 02447 South Korea; 2 Department of Internal Medicine, Kyung Hee University Hospital at Gangdong, College of Medicine, Kyung Hee University, 892 Dongnam-ro, Gangdong-gu, Seoul, 05278 South Korea; 3 Department of Surgery, Kyung Hee University Hospital at Gangdong, College of Medicine, Kyung Hee University, Seoul, 05278 South Korea; 4 Department of Anesthesiology and Pain Medicine, College of Medicine, Kyung Hee University, Seoul, 02447 South Korea

**Keywords:** Upper gastrointestinal bleeding, Need of interventions, 30-day mortality, Prediction, Scoring system

## Abstract

**Background:**

Risk scoring systems are used to evaluate patients with upper gastrointestinal bleeding (UGIB). We compared Glasgow-Blatchford score (GBS), modified GBS (mGBS), and Pre-endoscopy Rockall score (Pre-E RS) for immediate application without endoscopic findings in predicting the need of interventions and the 30-day mortality in patients with UGIB.

**Methods:**

Patients who visited the emergency room with UGIB from January 2007 to June 2016 were included. GBS, mGBS, and Pre-E RS were obtained for all patients. The area under the receiver-operating characteristic curves (AUC) was used to assess the accuracy of the scoring systems to determine the need for interventions and 30-day mortality. Also, we investigated the potential cutoff scores for predicting 30-day mortality and the need for interventions.

**Results:**

In predicting the need for interventions, GBS (AUC = 0.727) and mGBS (AUC = 0.733) outperformed Pre-E RS (AUC = 0.564, *P* < 0.0001). In predicting 30-day mortality, Pre-E RS (AUC = 0.929) outperformed GBS (AUC = 0.664, *P* < 0.0001) and mGBS (AUC = 0.652, *P* < 0.0001). Based on AUC analyses of sensitivities and specificities, the optimal cutoff mGBS and GBS for the need for interventions was 9 (70.71% sensitivity, 89.35% specificity) and 9 (73.57% sensitivity, 82.90% specificity) respectively, and optimal cutoff Pre-E RS for 30-day mortality was 4 (88.0% sensitivity, 97.52% specificity).

**Conclusions:**

GBS and mGBS are considered to be moderately accurate in making an early decision about the need of interventions in patients with UGIB. Pre-E RS is considered to be highly accurate in early detection of patients at high risk for 30-day mortality without endoscopic findings. In addition, we suggested potential cutoff scores to predict the need of interventions for GBS and mGBS, and 30-day mortality for Pre-E RS. Further studies are needed to confirm the clinical applicability of results.

## Background

Upper gastrointestinal bleeding (UGIB), a common medical emergency, is reported in the emergency room by 5% per year and accounts for 2~3% of hospitalization in developed countries [1]. Mortality for UGIB reportedly ranges from 2% to 15% and rebleeding is as high as 10–30% [2–4]. Therefore, proper stratification for patients with UGIB can help to identify candidates for interventions including blood transfusion, endoscopic treatment, and radiologic or surgical intervention. Moreover, this process helps to decrease the 30-day mortality.

The 2010 International consensus guidelines recommended early risk stratification using proven factors for the management of patients with UGIB [5]. The recent American College of Gastroenterology practice guidelines for managing patients with ulcer bleeding also recommended that a risk assessment be conducted to stratify patients into high and low risk categories, which may assist in initial decisions such as timing of endoscopy, time of discharge, and level of care [6]. Early risk stratification in the emergency department facilitates proper treatment as well as rapid and accurate triage. This is important for timely administration of lifesaving treatments to patients and for reducing high medical care costs.

Several scoring systems have been developed to evaluate patients with UGIB [7, 8]. Decisions about urgent endoscopy are important in the diagnosis and treatment for patients with UGIB. The Rockall score (RS) is not suitable for the decision about urgent endoscopy since it requires endoscopic results. Whereas, the Pre-endoscopy Rockall score (Pre-E RS) and the Glasgow-Blatchford score (GBS) are suitable because they require only clinical and laboratory data. Therefore, they could be applied immediately without endoscopic findings. In addition, a modified GBS (mGBS), which eliminated subjective criteria from GBS, has been introduced [9].

In this study, we determined the immediate applicability without endoscopic findings of the GBS, mGBS, and Pre-E RS systems in estimating the need for interventions and predicting the 30-day mortality in patients with UGIB. Also, we investigated the potential cutoff scores to predict the need of interventions and 30-day mortality in applicable scoring systems.

## Methods

### Study population

This is the retrospective study which was carried out at a single teaching center. The medical records were used to recognize patients who visited the emergency department with UGIB between January 2007 and June 2016.

Patients >18 years of age who visited the emergency department with symptoms and signs of UGIB from either variceal or nonvariceal source were involved in the study. Patients diagnosed with lower gastrointestinal bleeding were excluded. Patients stable enough for direct discharge from the emergency room had been recommended out-patient endoscopic examination within 2 days according to protocol; and patients who had not visited the hospital after discharge were excluded. All participations were informed of the study purpose and written consents were obtained following the requirements of the Institutional Review Board of Kyung Hee University Hospital at Gangdong. This study was approved by the Institutional Review Board of Kyung Hee University Hospital at Gangdong (KHNMC IRB 2016–07-026).

### Data collection

One junior doctor reviewed each subject’s hospital record including admission notes, discharge records, laboratory data, and endoscopic results to identify the presence of UGIB. Data on age, sex, medication use, drinking, smoking, pulse rate, blood pressure, physical exam findings, laboratory results, co-morbidities, and endoscopic results were collected from each patient. In addition, data concerning interventions including blood transfusion, endoscopic intervention, radiologic intervention, or surgery and 30-day mortality were collected.

### Treatment

The high dose proton pump inhibitor (Pantoloc® Takeda GmbH, Singen, Germany, 80 mg bolus once then 40 mg every 6 h) was administered to all subjects with UGIB intravenously. The terlipressin (Glypressin® Ferring Pharmaceuticals, Saint-Prex, Switzerland, 2 mg initially followed by 1 mg every 4 h) was administered to subjects who were considered to have variceal bleeding intravenously. Actions were taken for patients requiring interventions. Interventions is defined as medical or surgical procedures including as follows; blood transfusion, combination endoscopic hemostasis using dilute epinephrine injection plus second hemostasis modality (contact thermal, mechanical, or sclerosant), radiologic arterial embolization, or surgery. Endoscopic hemostasis was performed when Endoscopic findings were judged as Forrest classification Ia, Ib, or IIa, and radiologic intervention or surgery were performed when endoscopic hemostasis failed.

### Scoring systems

The GBS, mGBS, and Pre-E RS were calculated for each patient, as previously described (Table 1) [7–10]. The mGBS includes only quantitative factors of the GBS, which are blood urea nitrogen, hemoglobin (Hb), systolic blood pressure (SBP) and pulse. The Pre-E RS excludes endoscopic finding from RS. After the calculation of scoring systems involved in this study, Receiver-operating characteristic (ROC) curves were constructed to evaluate the accuracy of several scoring systems in predicting the need for interventions and 30-day mortality. In addition, based on the analyses for the area under the ROC (AUC), optimal cutoff levels for intervention and 30-day mortality were investigated.

**Table 1:** Scoring systems involved in this study

Scoring system	Clinical factors	Parameters	Score
mGBS	Pulse	≥ 100	1
	Systolic blood pressure (mmHg)	100–109	1
		90–99	2
		< 90	3
	Blood urea nitrogen (mg/dL)	19–22.3	2
		22.4–27.9	3
		28–69.9	4
		≥ 70	6
	Hemoglobin for male (g/dL)	12–12.9	1
		10–11.9	3
		< 10	6
	Hemoglobin for female (g/dL)	10–11.9	1
		< 10	6
GBS (includes four additional factors)	Hepatic disease	Present	2
	Cardiac failure	Present	2
	Melena	Present	1
	Syncope	Present	2
Pre-E RS	Age	< 60	0
		60–79	1
		≥ 80	2
	Shock	absent	0
	Heart rate	> 100	1
	Systolic blood pressure (mmHg)	< 100	2
	Comorbidity	Nil major	0
		IHD, CHF, other major	2
		RF, HF, Malignancy	3

*mGBS* modified Glasgow-Blatchford score, *GBS* Glasgow-Blatchford score, *Pre-E RS* Pre-endoscopy Rockall score, *IHD* ischemic heart disease, *CHF* congestive heart failure, *RF* renal failure, *HF* hepatic failure

### Statistical analyses

Descriptive analyses were conducted using SPSS (version 21.0; SPSS Inc., Chicago, IL, USA). Categorical variables were expressed by number and percentage occurrence frequency. Receiver-operating characteristic (ROC) curves were conducted, and the area under the ROC curve (AUC) with a 95% confidence interval was calculated with MedCalc (version 16.4.3, MedCalc, Mariakerke, Belgium). Estimates of sensitivity, specificity, positive predictive value, and negative predictive value with 95% confidence intervals (CIs) were obtained for each scoring system, and the ability to predict the need for interventions and the 30-day mortality was compared with MedCalc (version 16.4.3) using the method described by DeLong et al. [11]. The significance level for all analyses was set at *p* < 0.05.

## Results

### Patient characteristics

Table 2 shows the characteristics of subjects involved in the study. Of 590 patients, 215 (36.4%) were female; 166 (28.1%) were ≤60 years old and 133 (22.5%) were between 60 and 70 years of age. 337 patients (57.1%) had heart disease involving ischemic heart disease and cardiac failure in terms of comorbidity. 41 patients (6.9%) had liver disease including acute or chronic hepatitis and liver cirrhosis. The presenting symptoms were melena (*n* = 288, 48.8%), hematemesis (*n* = 147, 24.9%), hematochezia (*n* = 135, 22.9%), and syncope (*n* = 20, 3.4%). 202 patients (34.2%) were taking aspirin, 90 patients (15.3%) were taking warfarin, and 26 patients (4.4%) were taking non-steroidal anti-inflammatory drugs (NSAIDs). 449 patients (76.1%) received blood transfusion. 441 patients (74.8%) underwent gastroscopy within 24 h of presentation to the hospital. Gastroscopy was not performed in 2 patients because they were considered as low risk and unlikely to require endoscopic therapy. Endoscopic intervention and radiologic intervention were performed in 191 (32.4%) and 13 (2.2%) patients respectively, and any surgery to treat UGIB was not performed. The 30-day mortality was 4.2% (*n* = 25). The averages of GBS, mGBS, and Pre-E RS were 10.0, 9.4, and 2.0, respectively.

**Table 2:** Characteristics of included patients

	Patients (*N* = 590)	Percentage (%)
Gender		
Female	215	36.4
Age		
Less than 60	166	28.1
60 ~ 79	133	22.5
More than 80	291	49.3
History of disease		
Liver disease	41	6.9
Renal disease	3	0.5
Heart disease	337	57.1
Metastatic malignancy	6	1
Presenting symptoms		
Melena	288	48.8
Hematemesis	147	24.9
Hematochezia	135	22.9
Syncope	20	3.4
Medication		
No	272	46.1
Aspirin or other antiplatelet such as clopidogrel	202	34.2
Warfarin and other anticoagulant	90	15.3
NSAIDs	26	4.4
Time for endoscopic examination		
Less than 6 h	233	39.5
6 ~ 24 h	208	35.3
24 ~ 48 h	143	24.2
After 48 h	4	0.7
No endoscopy	2	0.3
Transfusion		
No	141	23.9
Yes	449	76.1
Hemostatic procedures		
No	386	65.4
Endoscopic intervention	191	32.4
Radiologic intervention	13	2.2
Surgery	0	0
30-day mortality		
Survival	565	95.8
Death	25	4.2

*NSAIDs* non-steroidal anti-inflammatory drugs

### Endoscopic findings

Table 3 lists the endoscopic findings of included patients. 235 patients (39.9%) were diagnosed with gastric ulcer. Of them, 114 patients underwent endoscopic hemostatic procedures and 9 patients underwent radiologic intervention, trans-arterial embolization. 49 patients (8.3%) were diagnosed with variceal bleeding and 31 patients of them underwent endoscopic variceal ligation or histoacryl injection. 38 patients (6.4%) were diagnosed with duodenal ulcer. Of them, 16 patients underwent endoscopic hemostatic procedures and 1 patient underwent radiologic intervention. 23 patients (3.9%) were diagnosed with Mallory-Weiss syndrome, and 8 patients of them underwent endoscopic intervention. 17 patients (2.9%) were diagnosed with Dieulafoy lesion. Of them, 14 patients underwent endoscopic intervention, and 3 patients underwent radiologic intervention. UGIB by malignancy was detected in 25 patients (4.2%) and 8 patients of them underwent endoscopic intervention. 12 patients (2%), who showed normal finding in gastroscopy, were diagnosed with obscure gastrointestinal bleeding. They underwent capsule enteroscopy later. 89 patients (15.1%) were diagnosed with gastritis, which was considered to cause UGIB by mucosal hemorrhage. So they did not undergo endoscopic and radiologic interventions.

**Table 3:** Endoscopic findings of included patients

	Patients (I = 590)	Percentage (%)
Normal	12	2
Gastritis	89	15.1
Gastric ulcer	235	39.9
Esophagitis	86	14.6
Esophageal or fundic varix	49	8.3
Duodenitis	1	0.2
Duodenal ulcer	38	6.4
Portal gastropathy	1	0.2
Mallory Weiss syndrome	23	3.9
Malignancy	25	4.2
Dieulafoy lesion	17	2.9
Others	12	2

Others: polyps 6 Schatzki Ring 2 Esophageal diverticulum 4

### The comparison of scoring systems for predicting the need of interventions and 30-day mortality without endoscopic findings

**Fig. 1:**
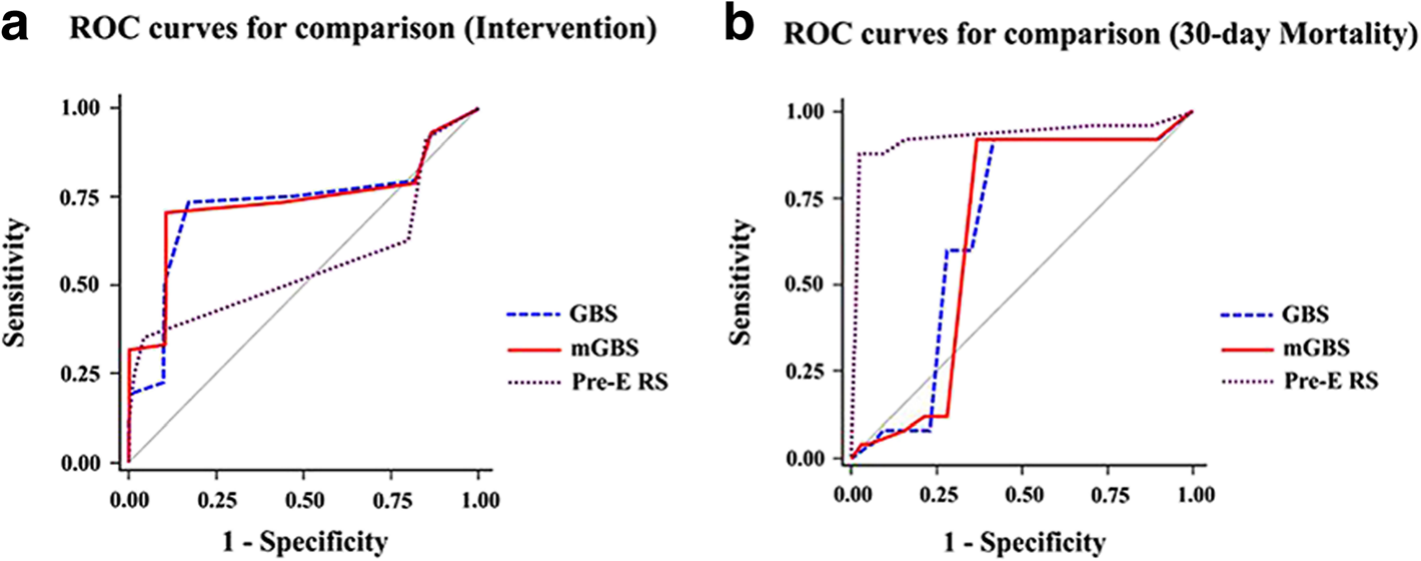
Comparisons of the GBS, mGBS, and Pre-E RS using AUC of each scoring system. **a** The need of interventions (blood transfusion, endoscopic, radiologic or surgical intervention). **b** 30-day mortality. ROC: Receiver-operating characteristic, AUC: Area under the receiver-operating characteristic curves, GBS: Glagow-Blatchford Score, mGBS: modified Glagow-Blatchford Score, Pre-E RS: Pre-Endoscopy Rockall score

Table 4 lists the patient numbers in each score and the patients numbers that underwent hemostatic interventions (transfusion and hemostatic procedures). Figure 1 presents the AUC of each scoring system for predicting the need of interventions and 30-day mortality. The GBS (AUC 0.727 [95% CI 0.689–0.762]) and mGBS (AUC 0.733 [95% CI 0.696–0.769]) outperformed the Pre-E RS (AUC 0.564 [95% CI 0.696–0.769], *p* < 0.0001) in predicting the need of interventions. There was not the significant difference between GBS and mGBS in predicting this point. In predicting 30-day mortality, the Pre-E RS (AUC 0.929 [95% CI 0.905–0.948]) outperformed the GBS (AUC 0.664 [95% CI 0.624–0.702], *p* < 0.0001) and mGBS (AUC 0.652 [95% CI 0.612–0.691], *p* < 0.0001).

**Table 4:** The patient numbers in each score and the patients numbers that underwent hemostatic interventions (transfusion and hemostatic procedures)

Score	GBS (Intervention Y/N)	mGBS (Intervention Y/N)	Pre-E RS (Intervention Y/N)
0	–	–	70 (Y = 24 / *N* = 46)
1	–	–	96 (Y = 80 / *N* = 16)
2	–	–	311 (Y = 77 / *N* = 234)
3	–	–	38 (Y = 29 / *N* = 9)
4	–	–	39 (Y = 35 / *N* = 4)
5	2 (Y = 1 / *N* = 1)	3 (Y = 1 / *N* = 2)	22 (Y = 22 / *N* = 0)
6	67 (Y = 27 / *N* = 40)	68 (Y = 28 / *N* = 40)	1 (Y = 1 / *N* = 0)
7	41 (Y = 26 / *N* = 15)	41 (Y = 27 / *N* = 14)	13 (Y = 12 / *N* = 1)
8	11 (Y = 9 / *N* = 2)	25 (Y = 12 / *N* = 13)	–
9	70 (Y = 4 / *N* = 55)	79 (Y = 5 / *N* = 74)	–
10	86 (Y = 21 / *N* = 65)	87 (Y = 33 / *N* = 54)	–
11	44 (Y = 34 / *N* = 10)	54 (Y = 52 / *N* = 2)	–
12	57 (Y = 19 / *N* = 38)	72 (Y = 4 / *N* = 68)	–
13	96 (Y = 55 / *N* = 41)	122 (Y = 80 / *N* = 42)	–
14	43 (Y = 13 / *N* = 30)	20 (Y = 19 / *N* = 1)	–
15	32 (Y = 31 / *N* = 1)	17 (Y = 17 / *N* = 0)	–
16	7 (Y = 6 / *N* = 1)	2 (Y = 2 / *N* = 0)	–
17	31 (Y = 31 / *N* = 0)	–	–
18	3 (Y = 3 / *N* = 0)	–	–
Total	590	590	590

Intervention Y: Number of patients using endoscopic intervention, radiologic intervention, surgery, and transfusion. Intervention N: Number of patient using only medication. GBS: Glasgow-Blatchford score, mGBS: modified Glasgow-Blatchford score, Pre-E RS: Pre-endoscopy Rockall score

Table 5 shows the ability to predict the need of interventions and 30-day mortality in applicable scoring systems. Based on AUC analyses of sensitivities and specificities in our study, the optimal cutoff mGBS and GBS to predict the need of interventions was 9 (70.71% sensitivity, 89.35% specificity) and 9 (73.57% sensitivity, 82.90% specificity) respectively. Of 397 patients with GBS > 9, 21 patients died in 30 day from presentation. Of them, 12 patients underwent endoscopy after 24 h from presentation, and 9 patients underwent endoscopy within 24 h (X^2^ = 21.675, *p* < 0.001). The odds ratio for 30 days mortality was 6.753 (95% CI, 2.729 to 16.712) meaning that the probability of death in the endoscopy performance over 24 h was 6.753 times higher than in the endoscopy performance within 24 h.

**Table 5:** Ability to predict need of interventions and 30-day mortality

Outcomes	System	Cutoff	Sensitivity, %	Specificity, %	PPV, %	NPV, %
Interventions	GBS	>6	93.21	13.23	49.2	68.3
	GBS	>7	79.64	18.06	46.8	49.6
	GBS	>8	75.36	52.26	58.8	70.1
	GBS	>9	73.57	82.90	79.5	77.6
	GBS	>10	61.43	86.45	80.4	71.3
	GBS	>11	50.71	89.68	81.6	66.8
	GBS	>12	36.07	90.00	76.5	60.9
	mGBS	>6	92.86	13.55	49.2	67.7
	mGBS	>7	78.93	18.06	46.5	48.7
	mGBS	>8	73.57	55.81	60.1	70.0
	mGBS	>9	70.71	89.35	85.7	77.2
	mGBS	>10	46.07	89.35	79.6	64.7
	mGBS	>11	33.21	89.68	74.4	59.8
	mGBS	>12	31.79	99.68	98.9	61.8
30-day mortality	Pre-E RS	>2	92.0	84.07	20.4	99.6
	Pre-E RS	>3	88.0	90.62	29.3	99.4
	Pre-E RS	>4	88.0	97.52	61.1	99.5
	Pre-E RS	>5	32.0	98.94	57.1	97.0
	Pre-E RS	>6	32.0	99.12	61.5	97.1

*PPV* positive predictive value, *NPV* negative predictive value, *GBS* Glagow Blatchford Score, *mGBS* modified Glagow Blatchford Score, *Pre-E RS* Pre-Endoscopy Rockall score

Of 372 patients with mGBS >9, 21 patients died in 30 day from presentation. Among them, 12 patients underwent endoscopy after 24 h from presentation, and 9 patients underwent endoscopy within 24 h (X^2^ = 19.380, *p* < 0.001). The odds ratio for 30 days mortality was 6.215 (95% CI, 2.510 to 15.390) meaning that the probability of death in the endoscopy performance over 24 h was 6.215 times higher than in the endoscopy performance within 24 h. In addition, based on AUC analyses of sensitivities and specificities in our study, the optimal cutoff Pre-E RS to predict 30-day mortality was 4 (88.0% sensitivity, 97.52% specificity).

## Discussion

In our study, the rate of subjects who received blood transfusion (76.1%) was higher compared with previously published studies [12, 13], because the rate of patients who showed hemoglobin under 8 g/dL was higher. On the other hand, 30-day mortality in our study was 4.2%, which was lower than that of previous report by Laursen et al. [12]. The reason of this difference was considered that the rate of UGIB caused by malignancy was lower in the present study. The other characteristics of patients such as the rate of patients who underwent endoscopic or radiologic interventions were consistent with the previous study [12].

Lin et al. [14] reported that cyclooxygenase-2 selective inhibitors (coxibs) significantly increased the incidence of UGIB, and various risk factors, including age, male gender, history of uncomplicated peptic ulcer disease, peptic ulcer bleeding, and *H. pylori* infection, contributed to the development of UGIB in coxibs users. The use of non-steroidal anti-inflammatory agents (NSAIDs) and anticoagulation agents associated with aging was considered to increase the development of UGIB by peptic ulcer. In our study, peptic ulcer disease including gastric ulcer and duodenal ulcer was the most common cause of UGIB, and 19.7% of patients took NSAIDs and anticoagulation agents. In our endoscopic findings, 2% of patients showing normal gastroscopic and colonoscopic findings were diagnosed with obscure gastrointestinal (GI) bleeding which is defined as persisting and/or recurrent GI bleeding of unidentified source after negative bidirectional endoscopic evaluation [15]. In case of obscure GI bleeding, we conducted capsule enteroscopy that is considered to be safe and effective for diagnosis of obscure GI bleeding [16].

Laine et al. [17] reported that patients who present with 6 to 12 h of the onset of UGIB symptoms show a significantly lower risk for transfusion owing to the higher hemoglobin level at presentation. On the other hand, patients delaying their presentation are more likely to have anemia, which increase transfusion requirement [17]. In the present study, we did not classify our study population according to time to presenation (rapid or delayed). Fortunately, Laine et al. [17] suggested that there is no apparent relationship between bleeding severity and time to presentation because there were no differences in mortality and requirement of hemostatic intervention with endoscopy, surgery or radiology among patients with rapid and delayed presentation.

When we planned this study, we considered a research involving only patients with non-variceal bleeding. However patients with symptoms and signs of UGIB who visited emergency department could not be distinguished clearly between variceal bleeding and non-variceal bleeding first. So, we analyzed patients with symptoms and signs of UGIB from either variceal or non-variceal source. However, when we analyzed data involving only patients with non-variceal bleeding, the result was similar with that of this study.

Several risk scoring systems have been produced to divide patients with UGIB into high- and low-risk categories. 2010 international consensus for UGIB recommended that the use of prognostic scales was needed for early stratification of patients with UGIB according to the degree of risk [5]. Early identification of patients who are likely to show the need of interventions or high mortality could improve efficiency of care. However, RS is not suitable for an early decision on need of urgent interventions in the management of patients with UGIB, since it requires endoscopic finding. For this reason, we compared 3 scoring systems (GBS, mGBS, and Pre-E RS) that could be applied immediately without endoscopic findings.

In this study, we used AUC to evaluate the 3 scoring systems for predicting the need of interventions and 30-day mortality in patients with UGIB. An arbitrary guideline proposed by Swets classifies scoring systems as non-informative (AUC = 0.5), less accurate (0.5 < AUC ≤ 0.7), moderately accurate (0.7 < AUC ≤ 0.9), highly accurate (0.9 < AUC < 1) and perfect tests (AUC = 1) [18]. AUC analyses in this study indicated that GBS and mGBS were moderately accurate (AUC = 0.727 and 0.733, respectively) and superior to Pre-E RS (AUC = 0.564) in predicting the need of interventions without endoscopic findings, consistent with prior studies. Cheng et al. [9] prospectively evaluated 199 patients with UGIB. They reported that mGBS (AUC = 0.85) performed as well as the GBS (AUC = 0.86, *p* = 0.81), and outperformed the Pre-E RS (AUC = 0.66*, p* < 0.0001) in predicting the need of interventions. Stanley et al. [19] reported that GBS was superior to Pre-E RS in predicting the need of interventions in a prospective study on 1555 subjects. On the other hand, the Pre-E RS is reported to be superior to GBS in predicting mortality of patients with UGIB [20]. Similarly, our study also showed that Pre-E RS outperformed GBS and mGBS in prediciting 30-day mortality. Many studies have reported that the RS was more closely associated with the probability of death than the chance of rebleeding [8, 21, 22]. Rockall et al. [23] reported that the RS was originally developed to predict the mortality rather than the prediction of rebleeding. The RS without endoscopy can identify the patients who are less likely to require intensive health care, and be used for endoscopic evaluation as outpatients, which improves the quality of patients’ care and allows substantial resource savings [24].

A recent retrospective study proposed that early gastroscopy should be performed for high-risk patients with GBS >12 to reduce mortality [25]. Our study indicated that the risk of significant bleeding to need interventions increased in patients with GBS > 9 or mGBS >9. Our results suggest that patients with GBS > 9 or mGBS >9 need hemostatic interventions and have to undergo early gastroscopy. Collectively, these findings advocate guidelines for early gastroscopy in high-risk patients with UGIB, which allow risk stratification and provision of appropriate endoscopic treatment [5]. Previous study showed that RS < 2 was indicative of low risk of mortality [10]. In our study, patients with Pre-RS > 4 showed high 30-day mortality, indicating that patients with Pre-RS > 4 should be managed with more immediate and intensive care to lower 30-day mortality. However, large prospective trials are needed for further evaluation of cutoff values.

This is the large-scale retrospective study to propose an appropriate scoring system without endoscopic findings for predicting the need of interventions and 30-day mortality in patients with UGIB. Our results may be useful to physicians in the emergency department for early decisions on interventions and prognosis. However, our study had some limitations. First, this was a single-center study from a teaching hospital, hence, our results cannot be generalized to all patients with the symptoms of UGIB. Second, there are generally some biases affecting the veracity of the retrospective study, such as selection bias, information bias and confounding, although all medical records used in this study were reviewed and entered into a database by one medical doctor for the quality of data. Finally, the rate of high-risk patients presenting with the symptoms of UGIB was high in this study. Therefore, further studies are required for accurate clinical applicability of results.

## Conclusions

The GBS and mGBS are considered to be moderately accurate with good sensitivity and specificity in making an early decision about the need of interventions in patients with UGIB. Pre-E RS is considered to be highly accurate in early detection of patients at high risk for 30-day mortality without endoscopic findings. In addition, we suggested potential cutoff scores to predict the need of interventions in GBS and mGBS and 30-day mortality in Pre-E RS. These risk scoring systems predicting the need of intervention and/or 30-day mortality can contribute to saving lives and alleviating the severity of a patient’s condition. Especially, these scores may be useful in case of emergency where endoscopy are not available. In this sense, the verification of risk scoring systems through the substantial clinical data conducted in this study may be beneficial to both physicians and patients. However, the perfect score should be applicable in pre- and post-endoscopy. Further studies are needed to confirm the clinical applicability of results.

## Abbreviations

AUC: Area under the receiver-operating characteristic curves
Cis: Confidence intervals
COX-2: Cyclooxygenase-2
Coxibs: cyclooxygenase-2 selective inhibitors
GBS: Glasgow-Blatchford score
GI: Gastrointestinal
Hb: hemoglobin
mGBS: modified GBS
NSAIDs: Non-steroidal anti-inflammatory agents
Pre-E RS: Pre-endoscopy Rockall score
ROC: Receiver-operating characteristic
RS: Rockall score
SBP: Systolic blood pressure
UGIB: Upper gastrointestinal bleeding
